# Evidence of CD1d pathway of lipid antigen presentation in mouse primary lung epithelial cells and its up-regulation upon *Mycobacterium bovis* BCG infection

**DOI:** 10.1371/journal.pone.0210116

**Published:** 2018-12-31

**Authors:** Zaigham Abbas Rizvi, Niti Puri, Rajiv K. Saxena

**Affiliations:** 1 School of Life Sciences, Jawaharlal Nehru University, New Delhi, Delhi, India; 2 Faculty of Life Sciences and Biotechnology, South Asian University, New Delhi, Delhi, India; Institut Pasteur, FRANCE

## Abstract

Presentation of a prototype lipid antigen α-Galactosylceramide (αGC) was examined on primary epithelial cells derived from mouse lungs and on bronchoalveolar lavage (BAL) cells that essentially comprise alveolar macrophages. Presence of CD1d molecules coupled to αGC was demonstrated on both types of cells pre-treated with αGC, suggesting that both cell types are equipped to present lipid antigens. Internalization of *Mycobacterium bovis* Bacillus Calmette–Guérin (BCG: a prototype pathogen), a pre-requisite to the processing and presentation of protein as well as lipid antigens, was clearly demonstrated in primary lung epithelial (PLE) cells as well as BAL cells. Both PLE and BAL cells expressed CD1d molecule and a significant up-regulation of its expression occurred upon infection of these cells with BCG. Besides CD1d, the expression of other important molecules that participate in lipid antigen presentation pathway (i.e. microsomal triglyceride transfer protein (MTTP), scavenger receptor B1 (SR-B1) and Saposin) was also significantly upregulated in PLE and BAL cells upon BCG infection. *In situ* up-regulation of CD1d expression on lung epithelial cells was also demonstrated in the lungs of mice exposed *intra-tracheally* to BCG. Taken together these results suggest that lung epithelial cells may have the ability to present lipid antigens and this pathway seems to get significantly upregulated in response to BCG infection.

## Introduction

Tuberculosis (TB) caused by *Mycobacterium tuberculosis* (Mtb), remains one of the deadliest diseases worldwide, in spite of tremendous advances in the understanding of host-pathogen interactions [1]. Lung provides the primary site of infection for Mtb, where the bacterium gains entry through the inhaled air [1,2]. Inside the alveolar spaces, macrophages interact with and respond to the invading pathogen [1]. Additionally, epithelial cells lining the alveolus are also exposed to pathogens and particles present in the inhaled air [3]. Recently we showed that BCG exposed PLE cells in culture are able to present antigens to isolated BCG sensitized CD4^+^ helper T cells [4]. Based on these results, we have suggested that the PLE cells could have a role in the generation of lung immunity to air-borne pathogens.

CD1 antigen presentation of lipid moieties is a parallel antigen presentation pathway that activates natural killer T (NKT) cells and complements the classical MHC II presentation pathway of T cell activation [5–8]. Lipid antigens derived from invading pathogens are presented in association with CD1 molecule and result in the induction of a rapid cytokine response by NKT cells that help generate an efficient immune response against fast mutating pathogens and cancerous cells [6–11]. Five distinct isoforms (CD1a-CD1e) of CD1 protein are expressed in humans but in mice, only one form (CD1d) is expressed [6–8,12,13]. CD1d is known to be expressed by professional antigen presenting cells (APCs) in mice. Intestinal epithelial cells in mice also express CD1d molecule and may participate in lipid antigen presentation [14]. *Mycobacterium* derived lipid antigens such as phosphatidylinositol mannosides have been shown to be presented by CD1d pathway [9,15–17]. CD1d lipid antigen presentation plays an important role in immunity to many pathogens and defects in CD1d pathway hinder development and maturation of NKT and T cells [18,19]. Furthermore, CD1d pathway disruption makes the system more prone to various viral and bacterial infections including Mtb infection in lungs [10,19–23]. CD1 mediated NKT response is also crucial for protective mucosal immunity and regulation of humoral immunity [24,25].

We have previously demonstrated CD1d expression on mouse lung epithelial cell line LA-4 and the ability of these cells to present prototype lipid αGC through CD1d pathway [26]. In the present study, we have extended this investigation to PLE cells obtained by digestion of lung tissue from mice. As a control, we also used BAL cells, that are rich in macrophages, as prototype professional APCs. Our results suggest that the PLE, as well as BAL cells, can present the prototype lipid antigen αGC. Both cell types can internalize BCG in culture and upregulate the expression of molecules involved in lipid presentation pathway, including the CD1d molecule. Lungs infected with BCG also have enhanced CD1d expression on epithelial cells. These results suggest that lung epithelial cells may participate in the induction of immunity to lipid antigens derived from airborne pathogens and that this pathway is up-regulated upon exposure of epithelial cells to BCG.

## Materials and Methods

### Animal handling and ethics statement

C57BL/6 inbred mice of age group 8–12 weeks were maintained in Jawaharlal Nehru University animal house facility with *ad libitum* water and mouse chow supply. The animals were housed under positive air pressure with 12 h light/ 12 h dark cycle at 25°C and 50% relative humidity conditions throughout the experiments. All experimental protocols requiring the use of animals were approved by the Jawaharlal Nehru University Institutional Animal Ethics Committee (IAEC, Registration No. 19/GO/ReBi/99/CPCSEA Dated:10.03.1999).

### Culture of BCG and staining

BCG (*M*. *bovis* Pasteur, TMCC no. 1011) was gifted by Ian Orm of the Microbiology department, Colorado State University, Fort Collins, CO. BCG was cultured in Sauton’s medium (with 0.05% Tween 80) in bio-shaker at 37°C for three weeks as previously described [4,27]. The bacterial cultures were harvested in the late log phase by centrifugation at 600 X g for 15 min. For labeling desired number of colony forming units (CFU) of BCG was suspended in PBS containing 10 μM Carboxyfluorescein succinimidyl ester (CFSE) (Sigma Aldrich, US) incubated for 1 h at 37°C on a rotisserie. The CFSE labeled BCG obtained were washed with PBS and analyzed on BD FACSCalibur or by confocal microscopy at 100X magnification (Olympus FluoviewTM–FV1000). All the experimental procedures involving BCG were carried out in Biosafety laboratory-2 (BSL-2) with prior approval of institutional biosafety committee.

### Isolation of mouse PLE and BAL cells

Isolation of lung epithelial cells from C57BL/6 mouse was performed as described previously by using the EasySep™ mouse epithelial cell enrichment kit (Cat. no. 19758; STEMCELL Technologies, Canada) [4,28–30]. Briefly, mouse lungs teased out of bronchiole, chopped into tiny pieces, were enzymatically homogenized, passed through a 40 μm cell strainer and then subjected to mouse epithelial cell isolation kit containing a cocktail of anti-CD31, anti-CD45, anti-TER119 antibodies following manufacturer’s protocol. Purity of the enriched lung cells were validated by staining with anti-B220, anti-CD3, anti-CD11b, anti-CD11c, anti-NK1.1 and F4/80 antibodies before (8.99%, 14.47%, 9.03%, 4.30%, 5.82% and 23.71% respectively) and after (0.28%, 0.53%, 1.09%, 1.15%, 0.15% and 1.48% respectively) enrichment. Recovery of PLE cells ranged from 4 X 10^6^ to 5 X 10^6^ with above 99% purity.

BAL cells were isolated by using a catheter-attached 1 ml syringe through a small transverse incision of the exposed trachea, as described before [4]. BAL cells were cultured overnight to enrich the adhering macrophage population, which was found to be 92.33% pure by F4/80 staining.

### Confocal microscopy

Primary cells were allowed to grow on glass coverslips in 12 well culture plates overnight (for BAL cells) or for 3 days (for PLE cells). Thereafter the cells were co-incubated with CFSE labeled BCG (MOI 100:1) for 4 h (for BAL cells) or for 24 h (for PLE cells) in 37°C incubator. The cells were then fixed with 4% paraformaldehyde (PFA) for 25 min at room temperature wells, washed and quenched twice with 50 mM NH_4_Cl (in PBS) for 5 min at room temperature. Coverslips were mounted onto slides using mounting medium and stored at 4°C in dark until acquiring the images by confocal microscopy (Olympus FluoviewTM–FV1000), the images were further analyzed and edited by using FV-10 ASW 1.7 Viewer software. Appropriate negative controls were taken to rule out false positives.

### Flowcytometric analysis

0.2x10^6^ cells were incubated with anti-mouse CD16/32 Fc block antibody (1 μg/10^6^ cells) for 20 min on ice prior to staining. Cells were then stained with PE-conjugated anti-mouse CD1d antibody (1B1 antibody) or their isotype control antibody for 30 min on ice and washed. For uptake studies mouse primary cells were cultured for 3 days (for PLE cells) or overnight (for BAL cells) and then co-cultured with CFSE labeled BCG (MOI 100:1) for 24 h at 37°C. The stained cells were analyzed on BD FACSCalibur by using CellQuest software by acquiring at least 10,000 events per sample.

### Immuno-histochemistry (IHC)

Mice were anesthetized and exposed to BCG (1 million CFU in 50 μl PBS) or phosphate buffer saline (PBS) vehicle by intra-tracheal instillation through aspiration as described before [2] and sacrificed 4 weeks later. Lungs were washed with PBS, suspended in 10% methyl formalin solution until further processing. The organs were then fine sectioned and preceded with hematoxylin and eosin (HE) stain alone or IHC staining. For IHC, staining was done by using rat anti-mouse CD1d primary antibody (1B1 antibody) followed by anti-rat IgG secondary antibody conjugated to HRP. The IHC stained section was then counter-stained with HE and observed under Ti Eclipse Nikon microscope at different magnifications. Appropriate experimental controls were also included in the study. The HE and IHC images were captured for at least 10 different fields with 3 replicates. Quantitation of CD1d staining was done by random blind scoring method.

### mRNA expression profiling

PLE and BAL cells were used for mRNA expression profiling studies through real time-PCR. Primers were designed for GAPDH [AAGCCCATCACCATCTTCCAG and AGACTCCACGACATACTCAGCA], CD1D [TCTCTGGCTTCTACCCAAAACC and ATCACCTCTGTGAGTACCCTGT], MTTP [GACAGCGTGGGCTACAAAATC and AGCTGTTATCGTGACTTGGATCAC], SR-B1 [ACTGCCTAACATCTTGGTCCTG and TCATCATCAGCTTCAGGCTCAC] and Saposin [CAGCACCAGTATTCCGAGGTCT & CAGCACACAGATTTCCTTGGGT] genes of *Mus musculus* using Primer-BLAST software (http://www.ncbi.nlm.nih.gov/tools/primer-blast/). RNA was isolated by using Trizol (TRI) reagent and was used for cDNA synthesis. Real-time mRNA expression profiling was performed by using cDNA samples obtained from mouse primary cell’s RNA by SYBR green method in a 96 well plate on 7500 RT-PCR system (Applied Biosystems, USA). The results obtained were normalized to GAPDH endogenous control for relative mRNA expression.

### CD1d antigen presentation assay

Antigen presentation assay for CD1d was carried out by already established assay model of using αGC as a lipid antigen and a monoclonal antibody (L363) capable of recognizing αGC:CD1d complex as previously described [26,31]. Briefly, cells cultured for overnight was co-incubated with 25 nM of αGC for 24 h. Thereafter cells were washed, stained with PE-conjugated L363 antibody and analyzed with a BD FACSCalibur flow cytometer using CellQuest software by acquiring at least 10,000 events per sample.

### Statistical analysis

All the experiments in the study were carried out at least 3 times independently. Student t-test was used to test for significance of differences between different sets of data using Sigma Plot software. Comparisons were considered significant at p ≤ 0.05.

## Results

### Presentation of lipid antigens by primary lung epithelial cells

**Fig 1:**
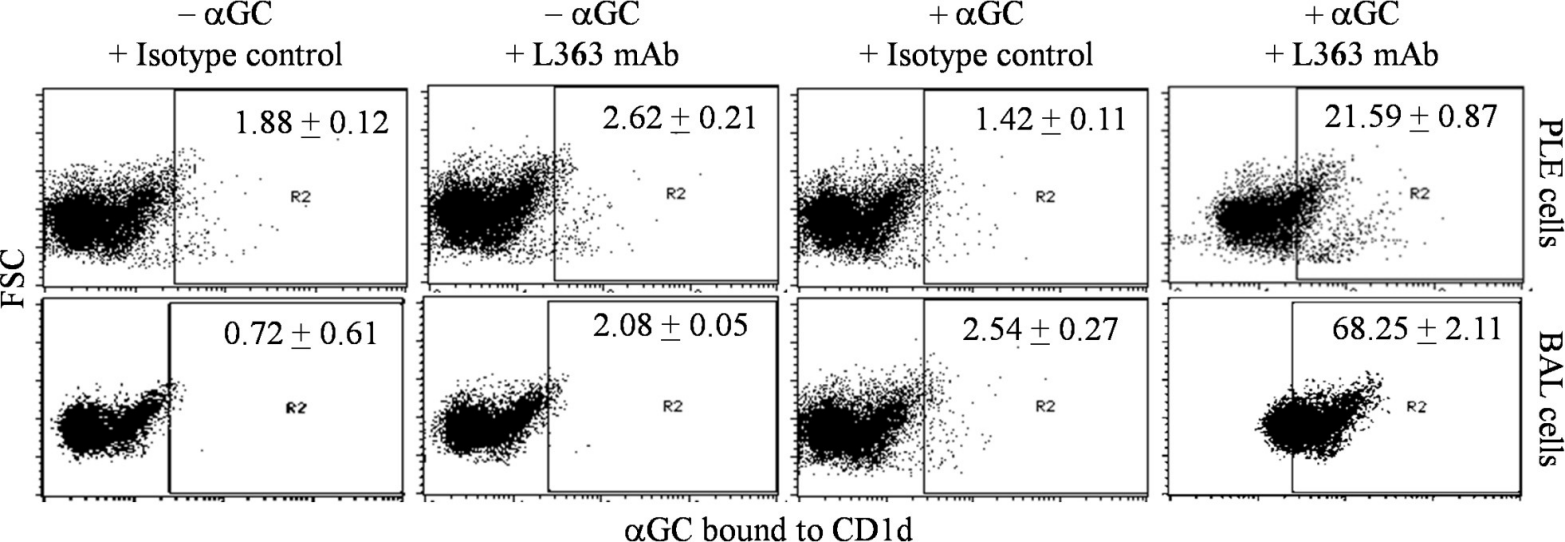
CD1d mediated antigen presentation of α-Galactosyl Ceramide (αGC) by primary cells of the lung. CD1d mediated antigen presentation of αGC by PLE and BAL cells were assayed by flow cytometry. 8x10^5^ cells were cultured overnight (for BAL cells) or for 3 days (for PLE cells) in 24 well plate and then incubated with 25 nM αGC lipid for 24 h at 37°C. Cells were washed, blocked by Fc block antibody followed by staining with isotype control or L363 antibody. Representative dot plots from 3 separate experiments with mean percent positive values ± SEM are shown. *p < 0.05 for significant difference.

We have previously shown that the LA-4 cells (a mouse lung alveolar epithelial cell line) could present αGC lipid moiety through the CD1d pathway *in vitro* [26]. We demonstrated this by using previously described αGC-CD1d model system of Yu *et al*. [31]. The model is based on specific recognition of αGC bound to CD1d molecule by the L363 antibody. The capacity of primary lung epithelial cells to present lipid antigens was however not demonstrated. In the present study, we used the αGC-CD1d model to assess the ability of PLE cells to present αGC lipid moiety through the CD1d pathway. Freshly isolated PLE cells from mouse lungs comprise a mixture of type I (podoplanin^+^CD74^-^) and type II (podoplanin^-^CD74^+^) epithelial cells. In cell culture, however, type II cells are lost and the surviving cells are essentially of type I by day 3 to 4 of culture [32,33]. PLE cells cultured for 3 days were used to assess αGC presentation. Fresh BAL cells, that essentially comprise alveolar macrophages, were also used in this study. Results in Fig 1 show that about 21.6% of the PLE cells pre-incubated with αGC were stained with L363 antibody, whereas only background staining was observed if αGC pre-incubation step was omitted. In BAL cells 68.2% cells were stained with L363 antibody. These results indicate that PLE cells, as well as BAL cells, had the ability to present αGC lipid through CD1d pathway but BAL cells were relatively more efficient presenters of the lipid antigen.

### Internalization of BCG by PLE cells in culture

**Fig 2:**
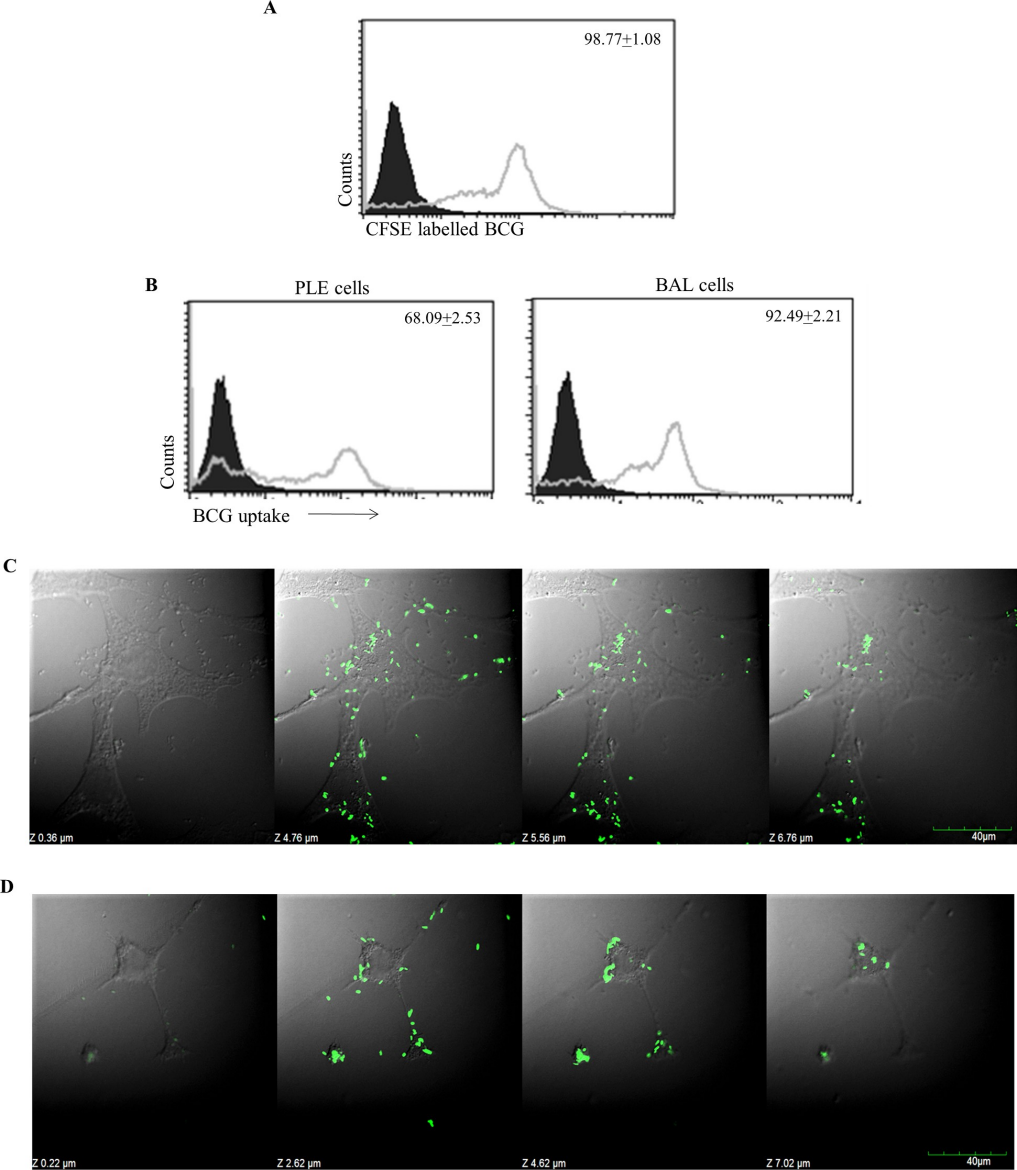
Uptake of BCG by PLE and BAL cells. Panel A shows the labelling of BCG with CFSE where more than 98% of BCG cells were labelled with the fluorescent dye (filled histogram—unlabeled BCG, open histogram CFSE labelled BCG). PLE and BAL cells were cultured with CFSE labelled BCG for 24 h (MOI 100:1) to study BCG uptake. Flow histograms in Panel B show the uptake of labelled BCG by PLE and BAL cells (filled histogram–control cells, open histograms–cells incubated with CFSE labelled BCG). Values for mean + SEM (3 experiments) of BCG positive cells are given within the histograms. Confocal microscopy and Z-sectioning to demonstrate intracellular BCG in PLE and BAL cells are shown in panels C and D respectively.

Owing to its anatomical location, lung epithelial cells lining alveoli as well as BAL cells present in the alveoli are exposed to air-borne pathogens entering the lungs [34,35]. Therefore, next, we examined the interaction of *Mycobacterium bovis* BCG with PLE and BAL cells. For this purpose, live BCG tagged with the fluorescent dye CFSE were incubated with PLE and BAL cells to study the uptake of BCG by these cells. Labeling of BCG by CFSE was found to be more than 98.8% (Fig 2A). Our uptake studies showed that 68% of the PLE cells incubated with fluoresceinated BCG were positive for BCG intake (Fig 2B, left panel) whereas 92.5% of the BAL cells were positive for BCG uptake (Fig 2B, right panel). Confocal microscopic examination and Z-sectioning further indicated that BCG was internalized by both PLE cells as well as BAL cells (Fig 2C & 2D respectively).

### Up-regulation of molecules involved in CD1d lipid antigen presentation in BCG infected PLE and BAL cells

**Fig 3:**
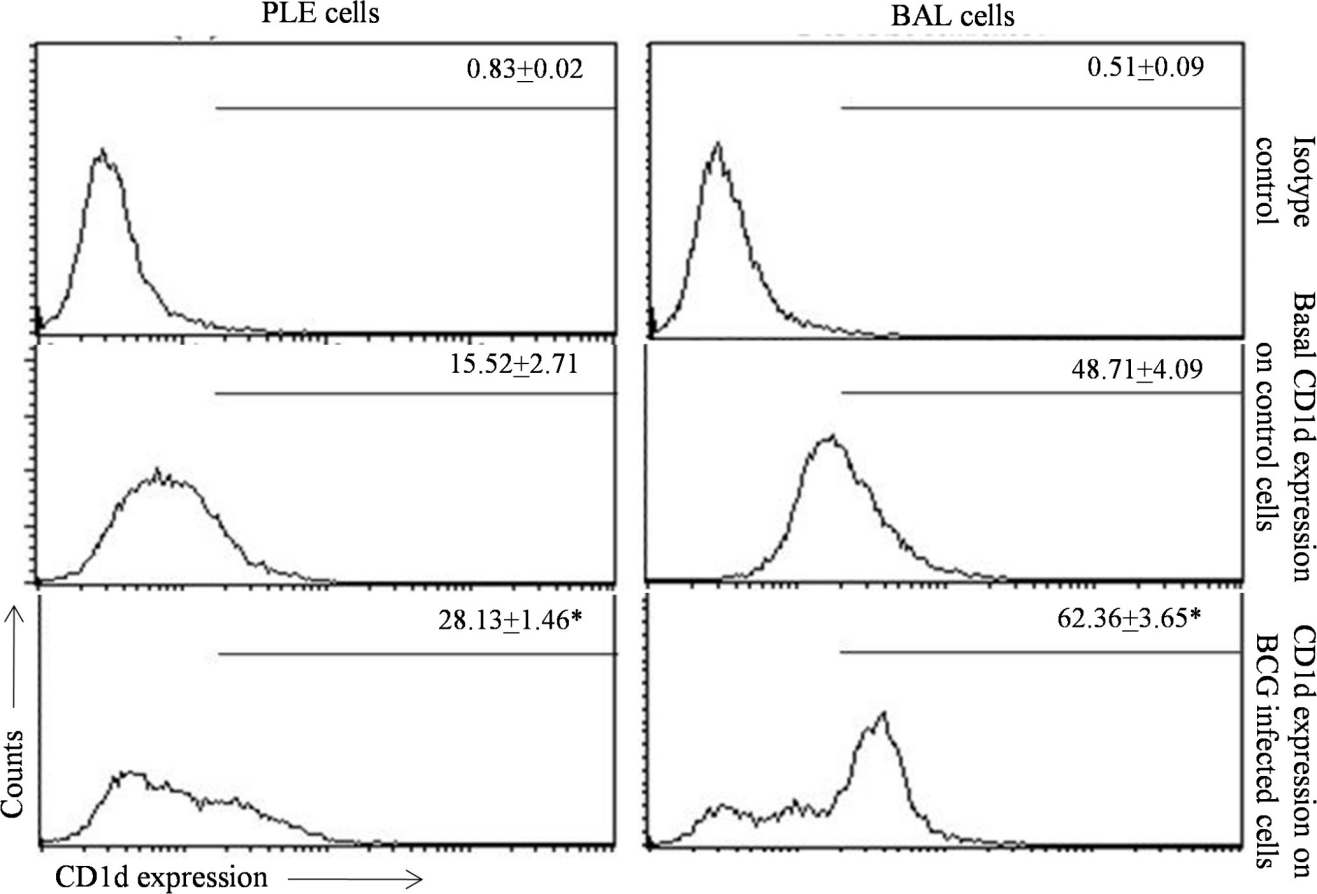
Cell surface expression of CD1d on control and BCG infected PLE and BAL cells. PLE and BAL cells were incubated with or without BCG (MOI 100:1 for 24 h) and CD1d expression were examined as described in Materials and Methods. Values in each panel indicate mean percent CD1d positive cells + SEM. *p < 0.05 for significant up-regulation of CD1d expression upon BCG infection.

**Fig 4:**
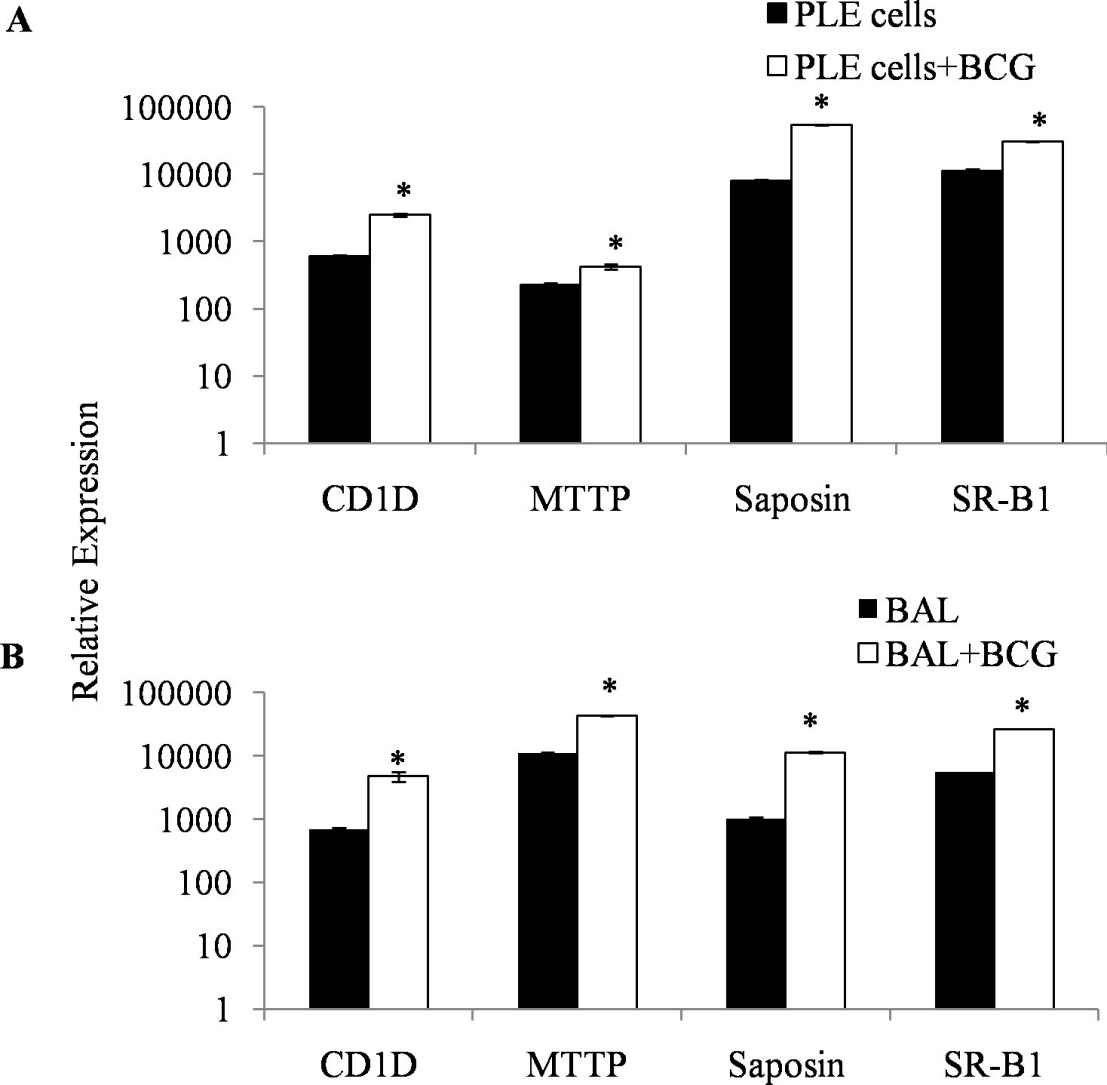
mRNA expression profiling of selected genes of CD1d pathway in PLE and BAL cells by real-time PCR. Quantitative RT-PCR was performed as described in Materials and methods in presence or absence of BCG infection (MOI 100:1 for 24 h). Closed bars represent the relative expression values for the 4 selected markers, for control, PLE (Panel A) or BAL (Panel B) cells, and open bars represent the corresponding relative expression values for BCG infected cells. Each bar graph is a representative of at least 3 independent experiments and the values shown represent the mean relative expression + SEM normalized to GAPDH control. *p < 0.05 for significant up-regulation of mRNA expression upon BCG infection.

The ability of PLE and BAL cells to present αGC lipid (Fig 1) was an indirect proof of the expression of CD1d molecule on these cells. We also examined directly the expression of CD1d and its modulation by BCG infection on PLE and BAL cells. PLE cells were found to express significant levels of CD1d molecule that was boosted 2 folds in BCG infected PLE cells (Fig 3). Similarly, significant up-regulation of CD1d (28% as compared to the basal expression) was also observed on BAL cells (Fig 3). Expression of some select crucial molecules like CD1d, MTTP, Saposin and SR-B1 involved in the uptake of lipid antigen and their processing was also examined on PLE and BAL cells by using quantitative real-time PCR. The relative quantitation values showed significant up-regulation for these molecules in PLE as well as BAL cells exposed to BCG (Fig 4).

### *In situ* expression of CD1d on lung epithelial cells

**Fig 5:**
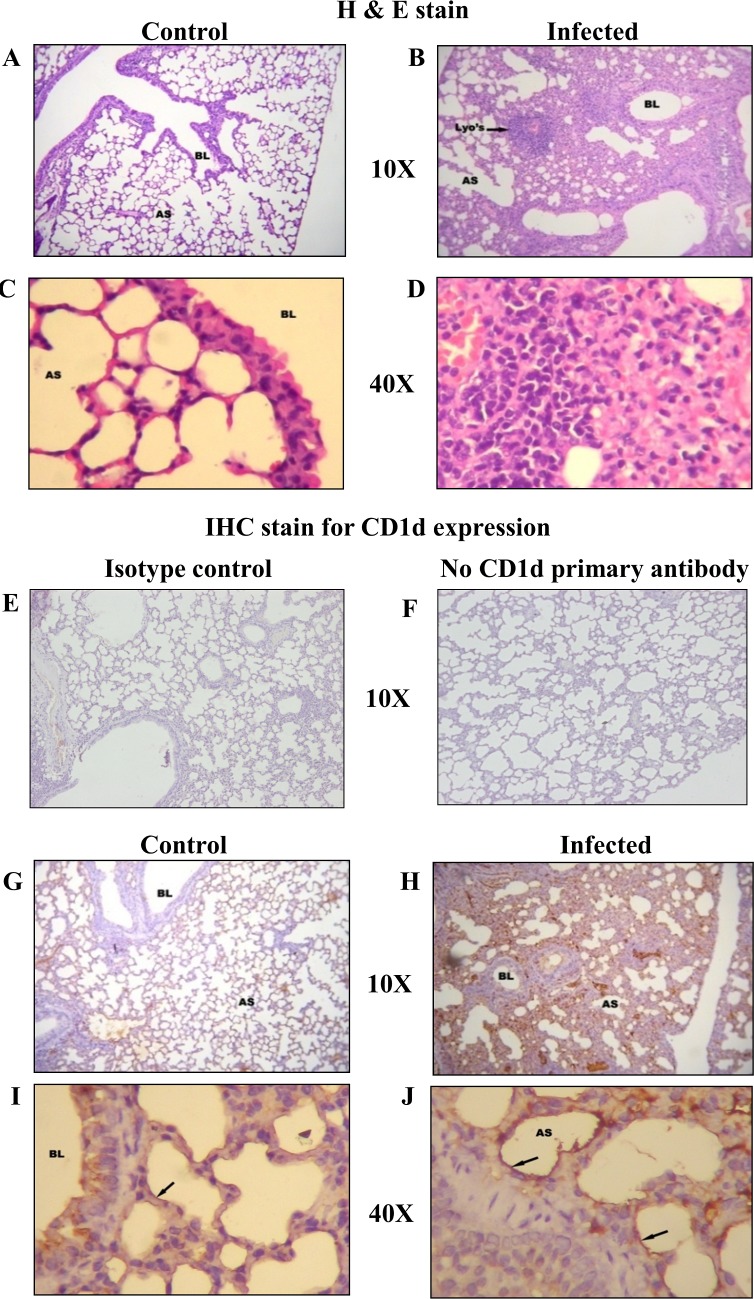
CD1d expression on lung cells *in situ* and its modulation by BCG infection. *In situ* expression of CD1d on the cells of the lung was studied by IHC as described in Materials and Methods. Panels A-D shows the HE staining of control and infected lungs. Left panels show control lungs at 10 and 40X magnifications. Right panels similarly show BCG infected lungs. Panels G-J shows the immunohistochemical (IHC) staining for CD1d staining in similar order. Panel E & F are the experimental controls for IHC using isotype control or “no CD1d primary antibody’. Within figure letters denote—Alveolar Space (AS), Terminal bronchiole (BL), lymphocyte-rich granuloma (Lyo’s), Arrows in panels I and J (←) indicate CD1d expression. Each image shown is a representative of at least 3 independent experiments.

While we were able to confirm the expression of CD1d molecule on PLE cells in culture, it was necessary to confirm whether CD1d is expressed *in situ* on lung epithelial cells. In lung sections stained with HE, the structure of the alveolus along with the epithelial cells lining the alveoli could be identified (Fig 5A–5D). To visualize the *in situ* expression of CD1d molecules on lung epithelial cells, IHC of the lung sections was carried out using a monoclonal anti-mouse CD1d antibody. An isotype control and a ‘no CD1d primary antibody’ was used as the experimental controls (Fig 5E & 5F). CD1d expression on the cells lining the alveolus could be clearly seen (Fig 5G–5J). In order to see if BCG infection modulates the CD1d expression, mice were administered BCG through intra-tracheal route. Results of HE staining of BCG infected lungs showed inflammatory changes and formation of granulomas (Fig 5B & 5D). Results in Fig 5J shows that the expression of CD1d on lung epithelial cells was up-regulated in lungs from BCG infected mice through random blind scoring method.

## Discussion

Airborne pathogens reach lung alveoli through the inspired air and come in contact with alveolar epithelial cells and alveolar macrophages. These two types of lung cells are thus amongst the very first host cells to interact with the airborne pathogens like *Mycobacterium tuberculosis*. Alveolar macrophages constitute a part of the immune system and are geared up to engulf the pathogen for subsequent processing and presentation of the antigens (derived from extracellular pathogens) for presentation to T lymphocytes. Alveolar macrophages can be isolated as BAL cells for studying their interactions with pathogens *in vitro*. Alveolar epithelial cells as such do not belong to the immune system but we recently showed that these cells have the ability to present mycobacterial antigens to sensitized T-helper cells *in vitro* and the efficacy of this process seems to be comparable to that seen with macrophages [4]. These results suggested that the alveolar epithelial cells may play a role in generating an immune response to mycobacteria and other airborne pathogens. Further, we also demonstrated that LA-4 lung epithelial cell line, as well as MH-S alveolar macrophage cell line, could also present lipid antigens through the CD1d lipid antigen presentation pathway [26]. In the present study, we further extended our study to determine if mouse PLE and BAL cells also have the ability to present lipid antigens through CD1d pathway.

Pathogen-derived lipid antigens associated with CD1d molecules are presented to NKT cells, that forms an important component of the immune response to pathogens [10,20,21,23]. This process may be examined by using a monoclonal antibody L363 that binds specifically with the CD1d-αGC complex (but not αGC or CD1d alone) that can be monitored using flow cytometry [26,31]. LA-4 and MH-S cells pre-incubated with αGC stained well with L363 antibody indicating that lipid antigens complexed with CD1d were expressed on these cells [26]. Using the same experimental design, we first demonstrated that αGC complexed with CD1d was present on both PLE cells as well as alveolar macrophages indicating that these cells too can present lipid antigens (Fig 1). CD1d molecules expressed on PLE and BAL cells could directly bind αGC that is a small (Mol. wt. 858) and highly hydrophobic lipid molecule and as such this binding does not require intracellular processing of the lipid moiety [36–38]. An important observation in our studies was that though basal cell surface expression of CD1d was around 15.52% on PLE cells, we could detect 21.59% positive PLE cells for CD1d-αGC complex. This greater percentage is attributed to α-GC induced upregulation of CD1d expression (S1 Fig).

With live pathogens, direct binding of lipid moieties would be difficult as the pathogens may not release such small molecular weight lipid moieties for surface binding on membrane expressed CD1d molecules. Internalization of the pathogens followed by processing of lipid antigens and complexing with CD1d may be required for lipid antigen presentation. While macrophages have this ability to engulf pathogens, we examined if PLE cells that are nonprofessional phagocytes too are able to internalize pathogens. We found that upon incubation with fluorescence-tagged BCG, both BAL and PLE cells could internalize BCG. Intracellular BCG was clearly demonstrable by a combination of flow cytometry and confocal microscopy. Thus in this study, we could demonstrate uptake of BCG by PLE and BAL cells as well as binding of a prototype lipid moiety αGC with CD1d molecules on these cells. It is, therefore, reasonable to infer that BAL, as well as PLE cells, are able to present lipid antigens bound to CD1d molecule. This is the first demonstration of CD1d presentation of lipid antigens in primary lung epithelial cells.

We further demonstrate that exposure to BCG results in a significant up-regulation of membrane expression of CD1d molecules on BAL and PLE cells (Fig 3). By using real-time PCR technique, we were also able to show that besides CD1d molecule, expression of other important molecules that are crucial in lipid antigen presentation pathway (MTTP, Saposin and SR-B1 molecules), are also up-regulated by exposure to prototype pathogen BCG. Of these molecules, Scavenger receptor class B 1 (SR-B1) facilitates the uptake of extracellular lipids [35], and microsomal triglyceride transfer peptide (MTTP) and Saposin are required for intracellular loading of lipid molecules on CD1d molecules [8,11,13,39–41]. Up-regulation of all these molecules upon exposure to pathogen suggests that pathogen exposure may prepare the cell for more efficient presentation of lipid antigens through CD1d pathway in order to generate a protective immune response.

Finally, we also looked for expression of CD1d molecule on lung epithelial cells *in situ* in lungs of control and BCG exposed mice. Mice exposed *intra-tracheally* to live BCG showed inflammatory changes in the lungs (accumulation of mononuclear cells and the presence of granulomas). By using the immune-histochemical technique and its quantitation through random blind scoring by un-related un-biased observers, we found a significant up-regulation of CD1d expression on lung epithelial cells lining the alveoli (S2 Fig).

MHC class I and II expression on healthy APCs requires the binding of peptides to the peptide-binding groove on these molecule, that happens intracellularly [42,43]. Without the binding of peptides, the MHC molecules do not properly fold and fail to be expressed on the membrane [43]. Likewise, CD1d molecules (which are structurally similar to MHC 1 molecules) fail to express without bound lipid moieties [5,13,44]. In normal uninfected APCs, CD1d molecules are expressed with endogenous lipids that get exchanged with lipids derived from pathogens in infected APCs [13,45]. We had previously shown that primary lung epithelial cells can present peptide antigens of BCG by using cytokine release from BCG sensitized T-helper cells as an assay [4]. Since lipid antigens are presented to NKT cells that do not require a prior sensitization and are present in very small numbers, it is difficult to directly demonstrate NKT activation by BCG derived lipids presented on CD1d molecules on the membrane of APCs. We, therefore, approached the issue in an indirect manner. First, we demonstrated that the primary lung epithelial cells express CD1d molecules and that these molecules can bind the prototype lipid antigen αGC. We further demonstrated that PLE cells can internalize BCG and that results in up-regulation of CD1d expression on the membrane and up-regulation of several important molecules involved in lipid antigen presentation pathway. Finally, up-regulation of CD1d was demonstrated on lung epithelial cells infected *in situ* by BCG. In all these experiments, BAL cells (alveolar macrophages) were used as positive control cells. Taking all these observations together, we infer that lung epithelial cells like the alveolar macrophages may have the ability to present lipid antigens through CD1d pathway. Since the presentation of peptide antigens by PLE cells was previously demonstrated [4] it seems that the PLE cells may be able to present both peptide and lipid antigens and may play an important role in the development of immune response to pathogens. Lung epithelial cells are strategically placed on the site of the first encounter with the air-borne pathogens and ability to present both antigenic peptides and lipids by these cells may confer an immunological advantage in the generation of a comprehensive immune response in lungs.

## Supporting information

S1 Fig Cell surface expression of CD1d on control and αGalCer treated PLE cells. PLE cells were incubated with or without αGalCer (25 nM αGC lipid for 24 h at 37°C) and CD1d expression was examined as described in Materials and Methods. Values in each panel indicate mean percent CD1d positive cells ± SEM. *p ≤ 0.05 for significant difference.
(TIF)

S2 Fig Quantitation of IHC staining for CD1d expression on PLE cells in lung section. Random blind scoring on the scale of 0–5 was done by unbiased, un-related observers for IHC staining for CD1d. 0 for the scale for set to the isotype control staining. The mean value of the independent scores given by 5 observers, un-related to the original experiment, were plotted in the form of bar graph+SEM. *p ≤ 0.05 for significant difference.
(TIF)
