# Supplementary figures and images for: Evidence of CD1d pathway of lipid antigen presentation in mouse primary lung epithelial cells and its up-regulation upon Mycobacterium bovis BCG infection
Source: PLoS One. 2018 Dec 31;13(12):e0210116. doi: 10.1371/journal.pone.0210116 (PMC6312317; doi:10.1371/journal.pone.0210116)

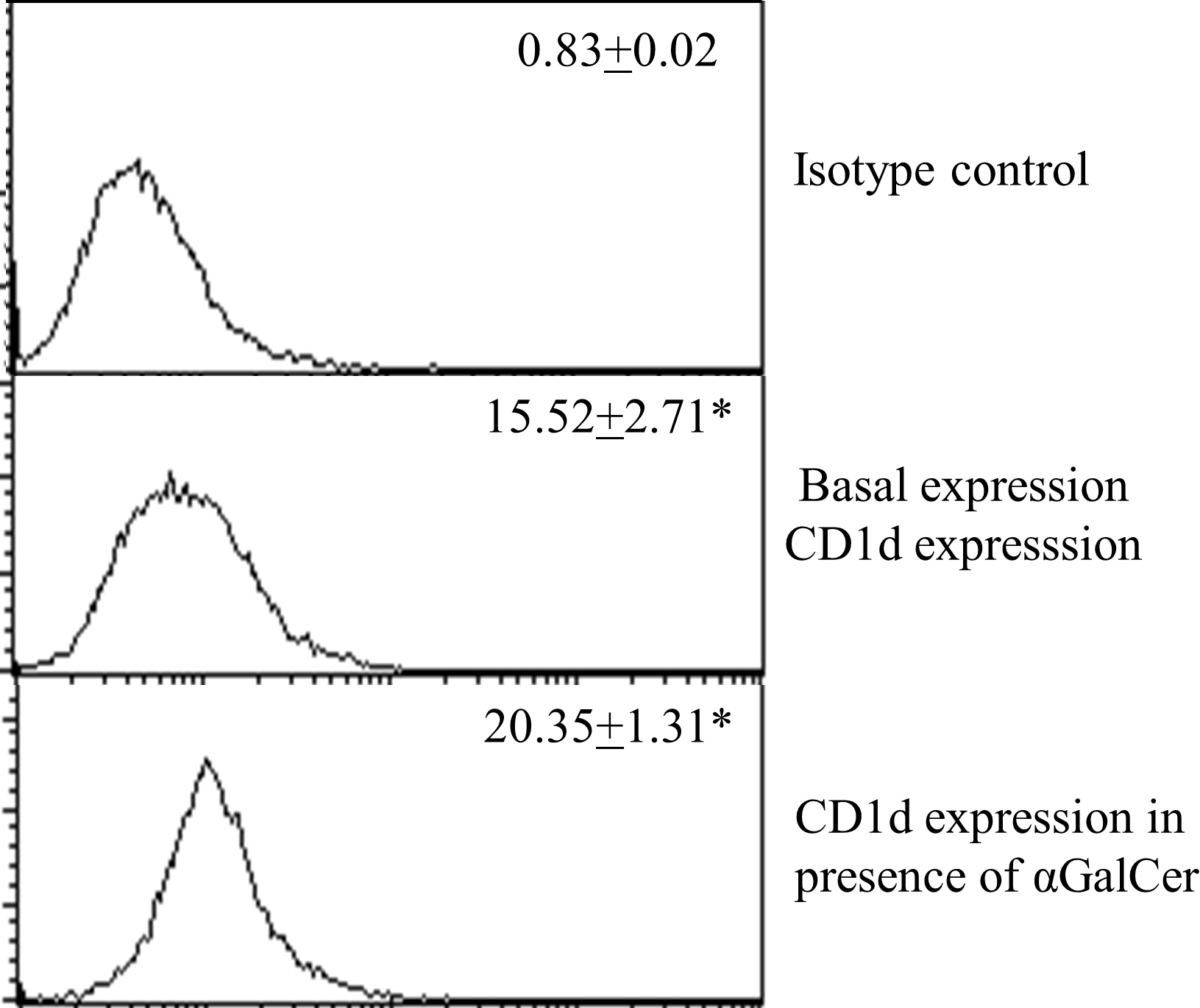

Supplement: S1 Fig — PLE cells were incubated with or without αGalCer (25 nM αGC lipid for 24 h at 37°C) and CD1d expression was examined as described in Materials and Methods. Values in each panel indicate mean percent CD1d positive cells ± SEM. *p ≤ 0.05 for significant difference. (TIF) [file pone.0210116.s001.tif]

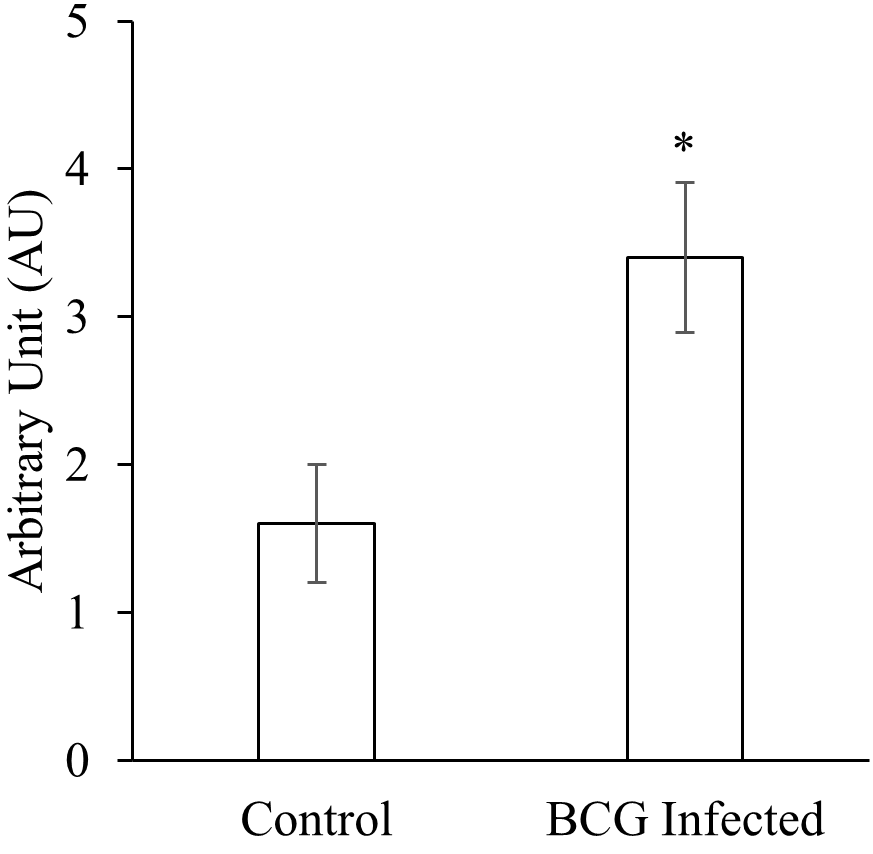

Supplement: S2 Fig — Random blind scoring on the scale of 0–5 was done by unbiased, un-related observers for IHC staining for CD1d. 0 for the scale for set to the isotype control staining. The mean value of the independent scores given by 5 observers, un-related to the original experiment, were plotted in the form of bar graph+SEM. *p ≤ 0.05 for significant difference. (TIF) [file pone.0210116.s002.tif]
